# Economic and Welfare Impacts of Providing Good Life Opportunities to Farm Animals

**DOI:** 10.3390/ani10040610

**Published:** 2020-04-02

**Authors:** Jessica E. Stokes, Siobhan Mullan, Taro Takahashi, Federica Monte, David C.J. Main

**Affiliations:** 1 Bristol Vet School, University of Bristol, Langford House, Langford BS40 5DU, UK; Siobhan.Mullan@Bristol.ac.uk (S.M.); taro.takahashi@bristol.ac.uk (T.T.); 2 School of Agriculture, Food and the Environment, Royal Agricultural University, Cirencester GL7 6JS, UK; David.Main@rau.ac.uk; 3 Sustainable Agriculture Sciences Department, Rothamsted Research, North Wyke, Okehampton EX20 2SB, UK; 4 Agriculture and Land Management, ADAS, Portview Road, Bristol BS11 9JE, UK; Fede.monte@adas.co.uk

**Keywords:** quality of life, positive experience, resource tiers, economic analysis, laying hens

## Abstract

**Simple Summary:**

An input-based framework to evaluate positive welfare opportunities for farm animals presents a case for incorporating quality of life measures into farm assurance schemes, thereby encouraging more producers to deliver higher welfare. Using an original dataset of UK laying hen farms that uniquely connects input-based measures of positive welfare to outcome-based measures of both positive and negative welfare, this study investigates the feasibility of evaluating positive welfare within certification schemes from both scientific and financial viewpoints.

**Abstract:**

Existing animal welfare standards for legislation and food certification programmes are primarily designed to avoid harms to the livestock, with minimal consideration given to their behavioural freedoms. Recent research has shown, however, that animal welfare should not only be evaluated by the absence of negative states but also by the presence of “good life” or positive experiences enjoyed by animals. The objective of the present study is to investigate the scientific validity and on-farm cost implications of utilising potential input-based measures of positive welfare as part of evaluation criteria for farm assurance schemes. Building upon the Farm Animal Welfare Council’s concept of good life opportunities, an assessment was undertaken on 49 noncaged laying hen farms across the UK by measuring on-farm resources to facilitate positive experiences alongside commonly measured metrics for welfare outcomes. The financial cost of providing these resources on each enterprise was also estimated using a farm-scale costing tool. The results suggested that 63% of resource needs that facilitate the behaviour opportunities of laying hens are already being provided by these producers, far above legal and commercial requirements. This practice attracts no reward mechanism or direct financial benefit under the current market structure. Additional provision of opportunities was positively associated with behavioural outcomes, but only limited impact was observed on health and productivity measures. Economic modelling indicated that significant room exists to further improve welfare scores on these farms, on average by 97%, without incurring additional costs. Together we argue that these results can be seen as evidence of market failure since producers are providing positive welfare value to society that is not being currently recognised. It is therefore contended that measuring and rewarding the supply of good life opportunities could be a novel policy instrument to create an effective marketplace that appropriately recognises high welfare production.

## 1. Introduction

Certification schemes for animal-originated food products provide an effective means to assure consumers of the farm’s compliance with welfare standards [1]. Traditionally founded on input-based assessment through measurements of resource provision [2,3] some recent programmes recognise the need for outcome-based assessment [4,5] and employ hybrid approaches that integrate information regarding on-farm resources, welfare outcomes and evidence of continuous improvement [6]. Beyond the primary purpose of consumer assurance, the analytical framework behind each certification scheme can also be utilised for on-farm decision support, scientific research as well as investigations into future legislative requirements [7].

Regardless of whether input-based or outcome-based, the majority of existing welfare-focused certification schemes are designed to reduce negative behavioural, health and physical outcomes on the farm by providing environments and management that are thought to safeguard the animal’s quality of life. While this method of certification holds a clear merit of excluding welfare-inconsiderate farms from supply chains, it has now been widely accepted that animal welfare should not only be evaluated by predominantly the absence of negative subjective states but also by the increasing presence of positive experiences [8,9]. This concept reflects the view that, in order to provide animals with good lives, it is essential to understand what they want as well as what they need to stay fit and healthy throughout their lifecycles [10].

Beyond the ethical perspective, there are multiple reasons why positive welfare should be considered as part of certification schemes. Improving positive welfare opportunities does not only enhance the animal’s living experiences but is also likely to reduce negative behavioural, health and physical outcomes [8], although the exact mechanism of this causal relationship is not well-understood. Furthermore, rewarding good outcomes is often considered to be a more effective method to induce farmers’ behavioural changes than penalising poor performances, as pride in stock is generally a stronger motivator than the desire to avoid difficulties arising from noncompliance [11]. These rewards also improve wellbeing of farmers through a higher level of job satisfaction [12,13,14], an oft-forgotten requirement to ensure long-term welfare of animals. Finally, evidence of the animals’ good life can add significant economic value to final products, as consumers are consistently shown to value positive welfare when appropriately informed [15].

Although it is recognised that increasing positive welfare is important, direct quantification of positive welfare involves complex challenges. Despite the considerable efforts made to identify suitable proxy measures for positive emotional state, e.g., through utilising the expressive quality of behaviour [16], there is little consensus as to how best to quantify positive welfare, particularly in a commercially feasible setting. While an increasing number of studies discuss positive welfare as a concept, evaluation methodologies have not progressed in any substantial manner [17] since Boissy et al. concluded that “there are as yet no feasible animal-based measures indicative of good welfare” [18]. Further validation and refinement are required to more effectively process on-farm information, including body language [19], vocalisation [20] and behavioural expressions such as play [21], before large-scale implementation of positive welfare measurements becomes a reality.

As an alternative approach, the Farm Animal Welfare Council (FAWC) proposed that positive welfare can, at least in part, be quantified by the level of provision of good life opportunities, or “resources that an animal does not need for biological fitness but are valued by the animal” [5]. Central to this concept is the conjecture that animals, like humans, value “variety to choose their preferred resource from” and, therefore, their welfare can be evaluated by the diversity of choice within their living environment. As resource inputs on the farm are more easily quantifiable and verifiable than the emotional state of animals, the adoption of this approach will likely result in a wider collection of objective evidence, a crucial prerequisite for incorporation of positive welfare assessment into private certification schemes and public policy intervention.

Motivated by this observation, the objective of the present study was to investigate the feasibility, concerning both scientific validity and potential cost implications for commercial farms, of utilising input-based measures of positive welfare as part of evaluation criteria for food certification schemes.

## 2. Material and Methods

Assessments of welfare-enhancing resource inputs and welfare outcomes were carried out on 49 noncaged laying hen farms in the UK recruited from the pool of members requiring an inspection between November 2013 and March 2014. With a current market share of 56% that is continuing to grow, the noncaged system is the most common egg production method in the country [22]. Pre-existing data were not available to guide a formal sampling strategy, although care was taken to include a diverse range of farms, concerning their location, environment, size, breed of birds and the scheme they participate in, so as to create a sample as closely representative of the industry as practical constraints permit (Supplementary Table S1). As per most noncaged farms in the UK, all sample farms were either a member of the RSPCA Assured (nonorganic) or Soil Association (organic) assurance schemes, which is an existing market requirement to ensure a price premium. Visits were made by five experienced scheme assessors and scheme advisors, all of whom were previously trained in the AssureWel outcome assessments for predominantly negative welfare [1]. Prior to data collection, they also attended an on-farm training session on the resource tier framework, during which the practicalities of flock assessment as well as the scoring criteria were discussed and standardised. All training sessions included feedback on consistency of assessment.

For input-based measures of positive welfare, the resource tier framework [23] was applied to each study farm. The framework consists of 13 resource needs categorised under five opportunities of comfort, pleasure, confidence, interest and healthy life (Table 1). For each resource need, farms were evaluated on a scale of 0 to 3 (no score, Welfare +, Welfare ++ and Welfare +++) based on physical resources available, on-farm environment and proactive management activities above what is stipulated by law [24] and codes of practice [25]. Depending on the category, the assessment was conducted by means of visual inspection, producer interviews or both (Table 1). The scoring system was designed to be additive across resource needs, and thus the maximum possible value for the total score, here labelled as good life score, was 39. As the original research [23] was solely designed to be a proof of concept study, this was the first time the framework was implemented for subsequent quantitative analysis.

For outcome-based measures of positive and negative welfare, six indicators commonly used by assurance schemes were collected on each farm (Table 2). An increase in these scores (feather loss, beak trimming, antagonistic behaviour, flightiness, mortality and litter score) represents a loss in quality-adjusted life expectancy of birds in the flock [1,16] and is therefore considered to be undesirable. For outcome-based measures of positive and negative welfare, qualitative behavioural assessment (QBA) was conducted by assessors on one study flock on all 49 farms using 15 descriptors originally developed by [26,27] and later adopted by the Welfare Quality protocol [16]. Following a flock observation of approximately five minutes, assessors used visual analogue scales to record a score for each descriptor. Principal Component Analysis (covariance matrix, no rotation) was used to derive components, the meaning of which were determined using the loadings of descriptor. Where more than one flock was present on the farm, the oldest flock was used for all welfare outcome assessments.

A detailed resource provision plan was created to match the conceptual “tiers” defined by [23] to actual resources required, which were subsequently linked to best-available price information, in British pound sterling (GBP) as at August 2019, to derive the total cost of interventions (Supplementary Table S2). To accurately represent the “tiers” concept of the framework, the cost structure for the three tiers within each resource need was designed to be incremental; in order to reach an upper tier, all resources required for lower tiers must also be present on the farm. This cost information was further combined with the results of on-farm assessments and, based on the actual scores awarded under each resource need, the outlay made by each farm to enhance positive welfare opportunities of animals was estimated. All costs were annualised and expressed as forgone net margins per dozen of eggs (~0.7 kg).

Following data collection, four patterns of correlations were examined using Pearson’s correlation coefficient (r): (1) amongst resource tier scores for five opportunities; (2) between resource tier scores and outcome-based measures; (3) between estimated costs and resource tier scores; and (4) between estimated costs and outcome-based measures. Pearson’s correlation coefficient was selected over Spearman’s rank correlation coefficient due to the cardinal (rather than ordinal) nature of the variables studied. Furthermore, in order to explore opportunities to reduce on-farm costs and encourage further adoption of higher welfare production by commercial producers, the relationship between a farm’s good life score and its total cost on welfare-enhancing resources was also investigated. Finally, estimated costs by sample farms were compared against the least cost, or the mathematically minimal outlay required to achieve the same good life score. In this comparison, the discrepancy between a farm’s actual expenditure and the derived least cost represented the degree of potential to improve the cost effectiveness of higher welfare production.

## 3. Results

### 3.1. Input-Based Measures

**Figure 1:**
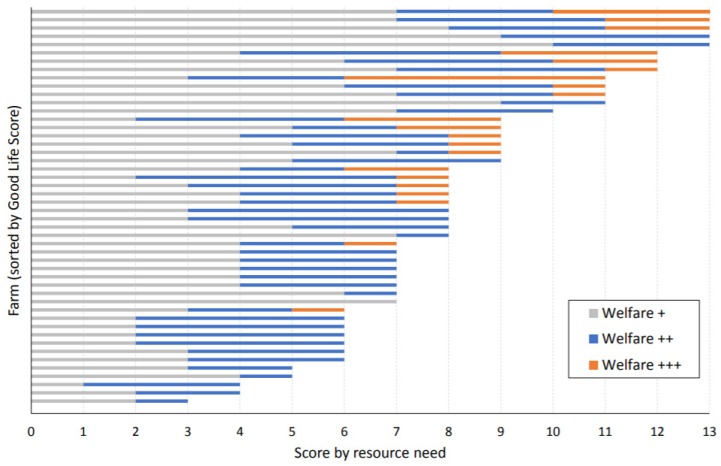
Number of resource needs (out of 13) achieved by each of 49 flocks.

**Figure 2:**
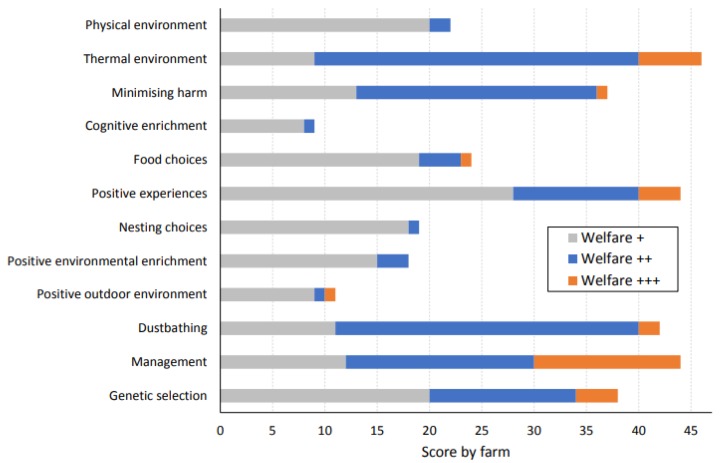
Number of flocks (out of 49) that achieve each of 13 resource needs.

Across 637 (49 × 13) combinations of flocks and resource needs, 63% achieved a score of Welfare + or above (Figure 1). A high degree of inter-farm variability was found within the flocks assessed; one farm scored no Welfare + or above under any resource need, while five satisfied all 13 resource needs at Welfare + or above. The proportions of farms satisfying higher tiers were also different across resource needs. For example, as many as 46 flocks (96%) marked Welfare + or above for social experiences, whereas only 9 flocks (18%) achieved Welfare + or above for cognitive enrichment (Figure 2). The result was similar at the upper end of the tiers, with 14 farms (29%) recording Welfare +++ for effective management, but no farms qualified at the same level under four resource needs (physical environment, cognitive enrichment, nesting choices and enriched environment). The maximum good life score is 39. This study found the average good life score across all sample farms was 12.6, with the range of 0–24.

### 3.2. Outcome-Based Measures

Feather losses at head/neck and back/vent areas were observed, respectively, amongst 9% and 10% of birds assessed on sample farms. Beak trimming was carried out, routinely before 10 days of age, at 37 farms (76%). Sixteen flocks (33%) had one or more instance of antagonistic behaviour, with 11 (22%) displaying aggressive behaviour and 5 (10%) observed to be feather pecking. Thirty-one flocks (63%) were recorded as calm, 14 (29%) as cautious and 4 (8%) as flighty. The median mortality of the previous flock was 5.4%, with the range of 2.6%–20%. Seventeen farms (35%) achieved the perfect litter score of 1, whereas fourteen (29%) recorded undesirable scores of 4 and above. Qualitative behavior assessment (QBA) assesses what animals feel in different situations. The QBA component that appeared to relate most closely to “mood” was the first component, which explained 50.4% of variance and had descriptors with loadings over 0.6 of Content, Calm, Happy at one end, and Depressed, Bored, Frustrated, Scared, Fearful, Distressed, Nervous, Tense, Agitated at the other. This component had a wide range of values between −2.59 and 1.55. As a standardised variable, however, these values are only informative in the context of within-sample comparisons.

### 3.3. Cost Structure

Across 13 resource needs, the average cost required to satisfy each tier (Welfare +, Welfare ++ and Welfare +++) was estimated to be 0.34, 0.55 and 1.21 GBP/doz, respectively (Supplementary Table S3). The estimated cost to achieve the perfect good life score (39) was 27.23 GBP/doz. Incrementally, some “upgrading”, or movement towards an immediately upper tier, was found to be significantly more cost effective than others. In particular, the marginal cost to achieve Welfare + under five resource needs, Welfare ++ under two and Welfare +++ under four were estimated to be less than 0.05 GBP/doz (Supplementary Table S4).

### 3.4. Correlation Analysis

Positive correlations were observed amongst resource tier scores for five opportunities recorded by the same flock, suggesting that farmers who create a positive welfare environment for animals tend to do so across multiple areas of farm management (Supplementary Tables S5 and S6). The good life score and the estimated total cost also showed a correlation (r = 0.822, *p* < 0.001), confirming that, the asymmetric cost structure notwithstanding, producers achieving positive welfare opportunities have generally invested more resources into the farm to improve the animals’ quality of life.

The correlation matrix between resource tier scores and outcome-based measures of welfare indicates that investment into on-farm resources is generally associated with reduction of negative outcomes *(*Table 3 and Supplementary Table S7*)*. Most notably, correlations were observed between comfort and flightiness (r = −0.383, *p* = 0.007), confidence and flightiness (r = −0.287, *p* = 0.046), as well as pleasure and beak trimming (r = −0.414, *p* = 0.003). The litter score was found to be negatively correlated with resource tier scores for all five opportunities, the good life score (r = −0.357, *p* = 0.012) and the estimated cost (r = −0.322, *p* = 0.024), suggesting that the litter condition may be a useful resource indicator of the overall level of animal welfare on the farm. Feather loss was not found to be associated with any score or cost variable.

The mood dimension score, an output-based measure of positive and negative welfare, was positively correlated with resource tier scores for all five opportunities as well as the good life score (r = 0.360, *p* = 0.011). The estimated cost was also positively correlated with the mood dimension score (r = 0.249, *p* = 0.084), suggesting that investment in on-farm resources may increase the likelihood of creating enhanced positive welfare outcomes for animals.

### 3.5. Least-Cost Strategy

**Figure 3:**
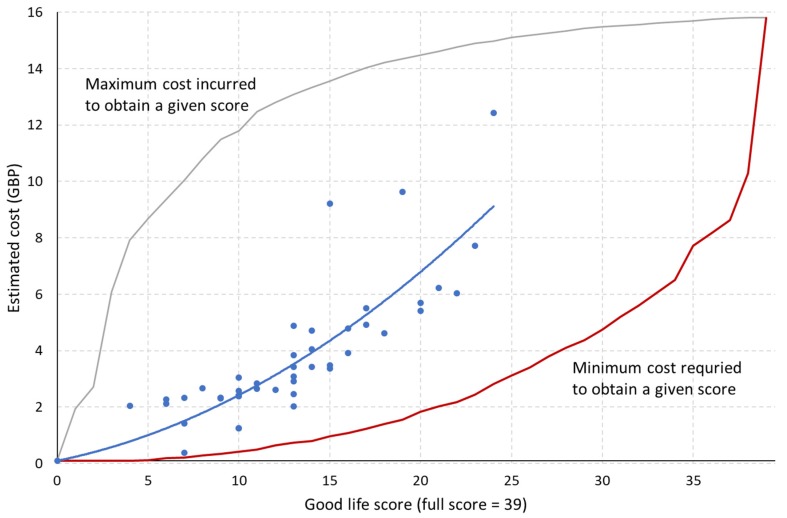
Relationship between resource tier score and estimated cost to achieve them at sample farms (*N* = 49). Quadratic trend curve suggests an exponential cost structure (y = 0.01x^2^ + 0.13x, *R*^2^ = 0.71), while the discrepancy between observed data (blue) and the minimum cost required to obtain a given score (red) shows the potential to reduce the expenditure without compromising the overall level of positive welfare.

A higher good life score was generally associated with a higher level of estimated investment. A closer investigation revealed, however, that this relationship was likely to be nonlinear (Figure 3), as the incremental cost to achieve Welfare ++ and Welfare +++ status tends to be higher than that to achieve Welfare + (Supplementary Table S4). The majority of sample farms were found to have spent considerably more on resources than theoretically required to achieve the same score (Figure 3), suggesting that significant room exists to further improve positive welfare opportunities without incurring additional costs. On average across 49 farms, the cost saving potential under the former approach was 81% of current total expenditures. Under the latter approach, the potential improvement in good life score was 12.2, or approximately twice the current average score.

## 4. Discussion

In this study the resource tier framework, a positive welfare scoring method assessing the resources which can provide good life opportunities of comfort, pleasure, confidence, interest and healthy life developed by the authors’ group [23], was applied on commercial laying hen farms located across the UK, creating a unique dataset linking input-based measures of positive welfare opportunity to outcome-based measures of both positive and negative welfare. Furthermore, the degree of investment currently being undertaken by producers to provide animals with good life opportunities was quantified and, based on these data, the efficacy of such investment vis-à-vis the predicted level of welfare status was examined. To the best of our knowledge, this is the first research exploring options to improve positive welfare of farm animals while explicitly considering their cost implications.

The above analysis revealed the extent to which farmers provide positive welfare opportunities that exceed current legal and commercial requirements. In total across the assessment of 13 resource tiers on all 49 flocks, 63% of assessments achieved a welfare + or above. Given that all sample farms are scheme-certified, these findings suggest that some farmers are providing positive welfare opportunities beyond what are required by law, code of practice and scheme guidelines and, crucially, not fully rewarded for these additional inputs. These good life opportunities, originally proposed by FAWC [5], were defined upon scientific evidence that additional resources are valued by animals even if they do not result in short-term changes in health and production parameters. Indeed, the derived relationship between resource tier scores and commonly assessed welfare outcomes indicated that provision of additional behavioural opportunities, while positively influencing the animal’s arousal (reduced flightiness) and mood (higher QBA score), was not associated with production performance (feather loss and mortality) of UK laying hen farms. Some may, therefore, be surprised to observe that a large proportion of farms are providing their stock with behavioural opportunities that do not necessarily contribute to their profitability; it is contended here that this result demonstrates the genuine interest held amongst commercial producers in providing an on-farm environment that promotes the animal’s positive experience. At the same time, the finding also indicates that good life opportunities should be seen as a complementary, rather than substituting, component of animal welfare, which would not be captured by existing legal requirements or outcome-based welfare assessments—such as the AssureWel animal welfare assessment that have been incorporated into certification schemes for the UK laying hen industry [1]. There was also significant variation between farms with five units achieving welfare + or above in all 13 resource tiers and one farm achieving welfare + or above in only three resource tiers. This means that the approach could be also used to promote continuous improvement toward higher welfare.

The positive correlation between mood dimension score with resource tier scores for all five opportunities as well as the overall good life score is consistent with [28], who reported a similar relationship on UK pig farms. As input-based methods are less likely to suffer from the assessor bias than outcome-based methods, the ability to use the former may provide a valuable solution to incorporate positive welfare assessment into large-scale certification schemes.

Given that some commercial producers are already providing positive welfare opportunities beyond existing requirements without any existing recognition or reward, their motives for doing so, and in particular nonfinancial incentives of providing additional resources, such as pride, social capital and value of animal wellbeing, warrant further investigation. If providing positive welfare resources is more motivating for farmers than making step changes to reduce negative welfare, a policy shift towards positive welfare may carry the potential to induce substantive human behavioural change throughout the supply chain [17]. It has previously been argued that animal welfare is a public good because it benefits the wellbeing of wider society beyond immediate stakeholders [29,30]. As an important recent example, the UK Agriculture Bill (https://services.parliament.uk/bills/2019-21/agriculture.html) proposes how farmers and land managers should be paid for public goods, including higher animal welfare standards. Under this concept, rewarding investment in good life opportunities could be a novel policy instrument to facilitate welfare improvement on the farm and, therefore, accumulation of social capital. This point is especially pertinent in light of the above result, namely that substantive production benefit may not exist for providing these opportunities.

In this context, perhaps the most important finding from the economic analysis was that significant opportunities lie ahead for producers to improve animal welfare without incurring further costs. The marginal cost to achieve at least some “good life opportunities”, i.e., welfare + across all five categories of resource needs was less than 0.05 GBP/doz. This suggests that there is a degree of market failure in the current market of high welfare products, which is preventing commercial farmers from rationally allocating resources to maximise the “production” of positive animal welfare. From the public economics perspective, this calls for research on the optimal mechanism of intervention, e.g., how to induce investment into resources of which marginal costs are lower than those currently supplied. With similar studies on negative welfare already advancing the knowledge in this area [31,32], such investigations could potentially lead to a “hybrid” approach, under which cost-effective provision of good life opportunities is combined with measures to reduce negative welfare outcomes.

Finally, it is worth recognising that appraising scientific validity of a welfare assessment method is a complex process [33], not least because there are numerous and often contradicting definitions of animal welfare [34]. The principal approach employed at the development of the resource tier framework was to safeguard content validity, or holistic inclusion of additional opportunities previously shown to be valued by animals [23]. It is noted however that the degree to which increasing levels of resource provision proposed by the current framework delivers an incremental increase in positive welfare requires further investigation. Validation in terms of both substantiate and demarcate the different levels of resource provision using positive behavioural outcomes should be employed. As outlined by FAWC [5], care must be taken before implementing this principle into product requirements, as provision of behavioural opportunities based on the animal’s desire could potentially be harmful. In the present study, however, there was little quantitative evidence to support that this was the case. If anything, good life opportunities were weakly associated with *reduced* mortality and, across five opportunities (comfort, pleasure, confidence, interest and health), no statistically significant case was detected where an improved opportunity was met by a reduced welfare outcome. Nonetheless, it is acknowledged that the limited sample size and relatively narrow scope of data collected relating to known risk factors for poor feather loss and other outcomes precluded a more detailed assessment of individual resources and their effects on outcomes and performance in this study. Also, the further inclusion of other key negative welfare outcomes such as keel bone damage, foot pad dermatitis, and health outcomes would facilitate a more comprehensive analysis. Further work to fully understand whether improving positive welfare opportunities can also reduce any negative behavioural, health and physical outcomes is required. To this end, additional work is being planned to further explore behavioural and health impacts of resources that appear to be particularly valued by animals.

In summary, this study has demonstrated that many UK laying hen farms are providing additional resources beyond that required by either legislation or certification requirements for which there was often no financial reward. Provision of these additional “good life opportunities” was positively associated with the mood dimension score, a behavioural outcome measure, but limited impact on health and productivity measures. Furthermore, economic modelling suggests that achieving good life opportunities across all five resource needs can be achieved with minimal additional cost.

## Figures and Tables

**Table 1:** Resource tier framework and methods of assessment.

Opportunity	Resource Need	Observations	Interviews
Comfort	Physical environment	√	
	Thermal environment	√	√
	Minimising harms	√	
Pleasure	Cognitive enrichment	√	√
	Food choices	√	√
Confidence	Positive experiences	√	√
	Nesting choices	√	
	Social experiences	√	
Interest	Enriched environment	√	√
	Positive outdoor environment	√	√
Healthy life	Dustbathing	√	
	Effective management		√
	Genetic selection		√

**Table 2:** Outcome measures used for validation of resource tier framework.

Type	Measure	Method	Source
Negative welfare	Feather loss	Number of birds, out of 50 samples randomly selected from the flock, with visible bare skin >5 cm in the head/neck and back/vent areas	[1]
	Beak trimming	Whether beak is routinely trimmed before 10 days of age (1) or not (0)	
	Antagonistic behaviour	Number of antagonistic behaviour (aggressive behaviour and injurious feather pecking) observed during the farm visit	
	Flightiness	Whether the flock is best described as flighty (2), cautious (1) or calm (0)	
	Mortality	Mortality rate of the flock immediately previous to that observed during the farm visit	
Resource outcome	Litter score	Condition of litter, as evaluated in the scale of 1–6: 1: Completely dry and friable; 2: Small moist/capped areas around drinkers/pop holes; 3: Large capped areas but sufficient space to dust bathe; 4: Largely wet or capped with few friable areas; 5: Largely capped or wet; 6: Largely wet or soggy.	[16]
Positive welfare	Mood dimension score	General “mood” of the flock, as expressed by the first principal component resulting from quantitative behavioural assessment	[16]

For negative welfare indices, a larger value indicates reduced animal welfare. For mood dimension score, a larger value indicates improved animal welfare.

**Table 3:** Correlation coefficients between resource tier scores and welfare outcome measures.

Opportunity	FL1	FL2	TRM	ANT	FLT	MRT	LIT	MDD
Comfort	0.121	−0.075	0.105	−0.222	**−0.383**	−0.217	**−0.350**	**0.297**
Pleasure	0.010	−0.008	**−0.414**	0.012	−0.134	0.149	−0.082	**0.287**
Confidence	0.020	−0.085	−0.224	−0.211	**−0.287**	−0.081	−0.226	**0.334**
Interest	0.080	−0.225	−0.066	−0.160	−0.208	−0.322	**−0.335**	0.249
Healthy life	0.177	0.012	−0.173	0.023	−0.219	−0.253	**−0.292**	0.193
Total score	0.124	−0.089	−0.200	−0.145	**−0.340**	−0.218	**−0.357**	**0.360**
Estimated cost	0.073	−0.061	**−0.356**	−0.158	−0.228	−0.086	**−0.322**	0.249

FL1: Feather loss (head and neck). FL2: Feather loss (back and vent). TRM: Beak trimming. ANT: Antagonistic behaviour. FLT: Flightiness. MRT: Mortality. LIT: Litter score. MDD: Mood dimension score. Bold values indicate *p* < 0.05; actual *p*-values are listed in Supplementary Table S7.

## Supplementary Materials

The following are available online at https://www.mdpi.com/2076-2615/10/4/610/s1, Table S1: Sample characteristics (N = 49), Table S2: Costs considered in economic analysis, Table S3: Total cost to satisfy each resource tier (£), Table S4: Incremental cost to satisfy higher resource tiers (£), Table S5: Correlation coefficients amongst resource tier scores and estimated cost, Table S6: *p*-values for correlations amongst resource tier scores and estimated cost, Table S7: *p*-values for correlations between resource tier scores and welfare outcome measures.
