# Supplementary material for: Economic and Welfare Impacts of Providing Good Life Opportunities to Farm Animals
Source: Animals (Basel). 2020 Apr 2;10(4):610. doi: 10.3390/ani10040610 (PMC7222722; doi:10.3390/ani10040610)
Supplement: Supplementary file 1 [file animals-10-00610-s001.pdf]

animals

Economic and Welfare Impacts of Providing Good Life Opportunities to Farm Animals †

Jessica E. Stokes [1,2,*], Siobhan Mullan [1], Taro Takahashi [1,3], Federica Monte [4] and David CJ Main [2]

[1] Bristol Vet School, University of Bristol, Langford House, Langford, Somerset BS40 5DU, UK; Siobhan.Mullan@Bristol.ac.uk (S.M.); taro.takahashi@bristol.ac.uk (T.T.)
[2] School of Agriculture, Food and the Environment, Royal Agricultural University, Cirencester, Gloucestershire GL7 6JS, UK; David.Main@rau.ac.uk
[3] Sustainable Agriculture Sciences Department, Rothamsted Research, North Wyke, Okehampton, Devon EX20 2SB, UK
[4] Agriculture and Land Management, ADAS, Portview Road, Avonmouth, Bristol, BS11 9JE, UK; Fede.monte@adas.co.uk
* Correspondence: jessica.stokes@rau.ac.uk

Supplementary Material

Table S1 Sample characteristics

Table S2 Costs considered in economic analysis

Table S3 Total cost to satisfy each resource tier

Table S4 Incremental cost to satisfy higher resource tiers

Table S5 Correlation coefficients amongst resource tier scores and estimated cost

Table S6 *p*-values for correlations amongst resource tier scores and estimated cost

Table S7 *p*-values for correlations between resource tier scores and welfare outcome measures

Table S1. Sample characteristics (N = 49).

Variable	Mean (range)	
Flock size (birds)	8,015 (110 – 16,000)	
Flock age (week)	42 (18 – 132)	
Breed	Lohmann Brown	26
	Hy-Line	5
	British Blacktail	7
	Warren	3
	Shaver brown	2
	Novogen breeds	3
	Other traditional breeds	3
Participation in certification scheme	RSPCA Freedom Food	39
	Soil Association	10

animals

MDPI

Table S2. Costs considered in economic analysis

Resource need	Welfare +	Welfare ++ [1]	Welfare +++ [1]
Physical environment	• Two types of litter substrates<br>• Two types of perches<br>• Labour cost for installation and maintenance	• Two types of perches for pellets<br>• Multiple flooring types during rearing	• Substrate flooring
Thermal environment		• Windbreaks<br>• Artificial shelters	• Pop hole covers
Minimising harms		• Ramps between pop holes, litter, slats, house and nest boxes	• Labour cost for extra monitoring of keel bone damages
Cognitive enrichment	• Log piles, fallen branches and fallen trees<br>• Labour cost for weekly replacement of enrichments	• Additional log piles, fallen branches and fallen trees<br>• Labour cost for weekly replacement of additional enrichments	• Puzzle feeders
Food choices	• Wholegrain oats provided separately from other feeds<br>• Insoluble grit provided separately from other feeds	• Feeders and drinkers on every level<br>• Pecking blocks<br>• Labour cost for scattering grain on litter	• Different feeders and drinkers on every level<br>• Chicory and clovers provided separately from other feeds
Positive experiences		• Labour cost for extra monitoring of flock experiences	• Labour cost for regular handling of birds<br>• Labour cost for handfeeding pullets
Nesting choices	• Extra nest boxes	• Enhanced substrates for nest boxes with wood shavings, buckwheat and oat husks	• Individual nest boxes
Social experiences	• Labour cost for managing pariah birds	• Visual barriers to create smaller groups	• Inclusion of cockerels<br>• Capital and labour costs for reducing stocking density

Enriched environment	• Alfalfa blocks, straw nets and pecking blocks<br>• Labour cost for placing and managing enrichments	• Alfalfa blocks, straw nets, pecking blocks for pellets<br>• Labour cost for placing and managing additional enrichments	• Projector and screen
Positive outdoor environment	• Trees and hedges to cover 5% of the range<br>• Artificial shelters immediately outside pop holes<br>• Roofed sandpits<br>• Animals (alpacas) kept on the range		• Establishment of an orchard to cover half the range
Dustbathing	• Enhanced litter with woodchip and sand<br>• Covered verandas<br>• Extra drinkers		• Deeper litter (15 cm)
Effective management	• Labour cost for weekly health and welfare outcome assessments	• Frequent health and welfare reviews with the vet (at each laying cycle)	• Participation in welfare initiatives
Genetic selection		• Reduced production as a result of welfare-focused selection	• Increased pullet cost for 'high maintenance' breeds

[1] In addition to all items considered for lower tiers

Table S3. Total cost to satisfy each resource tier (GBP)

Resource need	+	++	+++
Physical environment	0.27	0.98	1.12
Thermal environment	0.00	0.08	0.14
Minimising harms	0.00	0.08	0.09
Cognitive enrichment	0.30	0.60	0.62
Food choices	0.18	0.28	0.31
Positive experiences	0.00	0.07	0.23
Nesting choices	0.24	0.33	0.71
Social experiences	0.02	0.47	5.97
Enriched environment	0.68	0.91	0.95
Positive outdoor environment	0.77	0.77	0.98
Dustbathing	1.85	1.85	2.11
Effective management	0.07	0.28	0.34
Genetic selection	0.00	0.45	2.13

Values are foregone net margins per dozen eggs (~0.7 kg) compared to a production system with no welfare enhancement.

Table S4. Incremental cost to satisfy higher resource tiers (GBP)

Resource need	+	++	+++
Physical environment	0.27	0.71	0.13
Thermal environment	0.00	0.08	0.06
Minimising harms	0.00	0.08	0.01
Cognitive enrichment	0.30	0.30	0.02
Food choices	0.18	0.09	0.03
Positive experiences	0.00	0.07	0.16
Nesting choices	0.24	0.09	0.38
Social experiences	0.02	0.45	5.50
Enriched environment	0.68	0.23	0.04
Positive outdoor environment	0.77	0.00	0.21
Dustbathing	1.85	0.00	0.27
Effective management	0.07	0.21	0.06
Genetic selection	0.00	0.45	1.68

Values are foregone net margins per dozen eggs (~0.7 kg) compared to the resource tier one level below.

animals

MDPI

Table S5. Correlation coefficients amongst resource tier scores and estimated cost

Opportunity	COM	PLE	CON	INT	HEA	TOT	COS
Comfort	1						
Pleasure	0.422	1					
Confidence	0.504	0.612	1				
Interest	0.628	0.536	0.383	1			
Healthy life	0.303	0.370	0.401	0.338	1		
Total score	0.741	0.739	0.787	0.719	0.724	1	
Estimated cost	0.452	0.664	0.791	0.521	0.596	0.822	1

COM: Comfort. PLE: Pleasure. CON: Confidence. INT: Interest. HEA: Healthy life. TOT: Total score. COS: Estimated cost. All values are $p < 0.05$; actual *p*-values are listed in Supplementary Table S6.

Table S6. *p*-values for correlations amongst resource tier scores and estimated cost

Opportunity	COM	PLE	CON	INT	HEA	TOT	COS
Comfort	0						
Pleasure	0.003	0					
Confidence	<0.001	<0.001	0				
Interest	<0.001	<0.001	0.007	0			
Healthy life	0.034	0.009	0.004	0.018	0		
Total score	<0.001	<0.001	<0.001	<0.001	<0.001	0	
Estimated cost	0.001	<0.001	<0.001	<0.001	<0.001	<0.001	1

COM: Comfort. PLE: Pleasure. CON: Confidence. INT: Interest. HEA: Healthy life. TOT: Total score. COS: Estimated cost.

Table S7. *p*-values for correlations between resource tier scores and welfare outcome measures

Opportunity	FL1	FL2	TRM	ANT	FLT	MRT	LIT	MDD
Comfort	0.407	0.609	0.474	0.125	**0.007**	0.210	**0.014**	**0.038**
Pleasure	0.948	0.958	**0.003**	0.935	0.358	0.393	0.575	**0.046**
Confidence	0.893	0.561	0.121	0.146	**0.046**	0.645	0.118	**0.019**
Interest	0.584	0.120	0.653	0.272	0.152	0.060	**0.019**	0.084
Healthy life	0.223	0.937	0.235	0.877	0.131	0.143	**0.042**	0.184
Total score	0.397	0.541	0.169	0.320	**0.017**	0.208	**0.012**	**0.011**
Estimated cost	0.617	0.677	**0.012**	0.277	0.115	0.622	**0.024**	0.084

FL1: Feather loss (head and neck). FL2: Feather loss (back and vent). TRM: Beak trimming. ANT: Antagonistic behaviour. FLT: Flightiness. MRT: Mortality. LIT: Litter score. MDD: Mood dimension score. Bold values indicate $p < 0.05$. Corresponding correlation coefficients are listed within the main article (Table 3).
